# Improving the Utility of the Tox21 Dataset by Deep Metadata Annotations and Constructing Reusable Benchmarked Chemical Reference Signatures

**DOI:** 10.3390/molecules24081604

**Published:** 2019-04-23

**Authors:** Daniel J. Cooper, Stephan Schürer

**Affiliations:** 1 Department of Molecular and Cellular Pharmacology, Miller School of Medicine, University of Miami, Miami, FL 33136, USA; djc183@med.miami.edu; 2 Center for Computational Science, University of Miami, Miami, FL 33136, USA

**Keywords:** Tox21, high-throughput screening, FAIR data, data standards, ontologies, signatures, benchmarking, metadata

## Abstract

The Toxicology in the 21st Century (Tox21) project seeks to develop and test methods for high-throughput examination of the effect certain chemical compounds have on biological systems. Although primary and toxicity assay data were readily available for multiple reporter gene modified cell lines, extensive annotation and curation was required to improve these datasets with respect to how FAIR (Findable, Accessible, Interoperable, and Reusable) they are. In this study, we fully annotated the Tox21 published data with relevant and accepted controlled vocabularies. After removing unreliable data points, we aggregated the results and created three sets of signatures reflecting activity in the reporter gene assays, cytotoxicity, and selective reporter gene activity, respectively. We benchmarked these signatures using the chemical structures of the tested compounds and obtained generally high receiver operating characteristic (ROC) scores, suggesting good quality and utility of these signatures and the underlying data. We analyzed the results to identify promiscuous individual compounds and chemotypes for the three signature categories and interpreted the results to illustrate the utility and re-usability of the datasets. With this study, we aimed to demonstrate the importance of data standards in reporting screening results and high-quality annotations to enable re-use and interpretation of these data. To improve the data with respect to all FAIR criteria, all assay annotations, cleaned and aggregate datasets, and signatures were made available as standardized dataset packages (Aggregated Tox21 bioactivity data, 2019).

## 1. Introduction

The Toxicology in the 21st Century (Tox21) compound screening project is a collaborative effort by the National Institutes of Health (NIH), the Environmental Protection Agency (EPA), and the Food and Drug Administration (FDA) to develop and utilize new toxicity screening assays to examine potential detrimental effects to human health and biological processes [1,2,3,4]. The project tests approximately 10,000 environmental toxins for phenotypic effects in human metabolic processes through the use of gene-reporter systems [3]. Data produced through the Tox21 program and the compound library they built have been utilized for numerous predictive assays, including external examination of constitutive androstane receptor (CAR) [5], mitochondrial function [6,7], androgen receptor [8,9], and predictive data for in vivo toxicity and side effects in humans [10,11,12,13,14,15]. While these data have been produced, used, and reused in varied forms, it remains left to the individual analysis personnel to determine the best course to aggregate and clean the published Tox21 datasets for statistical analysis and reuse, thereby potentially limiting its impact.

To that end, we sought to improve the overall FAIR (Findability, Accessibility, Interoperability, and Reusability) compliance of the Tox21 datasets [16]. Initial publication and accessibility of the Tox21 data [17] represents substantial but relatively disparate data in addition to individual PubChem entries for assays. Individual assay information must be examined for key information and identifiers such as species, cell type, reporter type, and the exact protein/pathway affected. Reporting methods for assay data also differ, and key quality control data for compound batch purity are not included in the major PubChem releases. More and more, members of the biomedical community at large are seeking to improve data FAIRness by leveraging existing data standards, establishing new ones, and implementing substantial data curation efforts [18,19,20], among many other measures.

The Tox21 data in particular have potential for integrative analysis due to the nature of the reporter gene paradigm as well as the extent of the data produced and its characteristic of a dense matrix. Proteomics, transcriptomics, metabolomics, and target-based cell and biochemical screening data can have compatible metadata enabling their integrative analysis. We recently illustrated best practices of metadata management in another large scale data generation project [21], the Library of Integrated Network-based Cellular Signatures (LINCS) [22]. To that end, we endeavored to further improve the reusability of the Tox21 data and illustrate newfound usability after fully annotating assay information by established reference ontologies followed by aggregating the data to enable specific actionable insights. In this study, we performed three primary feats: (1) annotating the datasets utilizing the vocabulary provided in the BioAssay Ontology (BAO) [23,24,25,26] and other ontologies, (2) data cleaning (including filtering bad records and aggregating results by unique chemical compounds) and creating interpretable categories including reporter-specific and cytotoxicity outcomes to improve interoperability/integration, reusability, and facilitate analyses, and (3) illustrate re-use of the extensively annotated Tox21 datasets by analyzing promiscuity and selectivity of individual compounds and chemotypes. We examined the reported pAC_50_ values of the Tox21 reporter gene assay confirmatory datasets alongside the assays’ toxicity screen pairings for significance and sought to make the annotated, readied data more easily accessible and usable. The annotated and aggregated datasets are available via the LINCS Data Portal (LDP) [27] with a unique global resolvable dataset ID [28].

## 2. Results

### 2.1. Data Annotation and Categorization

To improve FAIR principle compliance, all 68 assays were manually curated and annotated based on the BioAssay Ontology vocabulary for key factors associated with Findability, Interoperability, and Reusability. Some annotations for Tox21, as well as other EPA and FDA projects and assays, are available on the ToxCast Dashboard [29] already. Here, we focused on ontology-centric terms and controlled vocabulary to better enable machine readability and data integration in addition to having the advantage of a single dataset download. Annotation included both generalized categorizations (including cell organism, screening campaign stage, and reporter type) and key identifier-based categorizations (including assay ID, precursor cell ID, and Uniprot ID of modified protein for reporting). All annotations relating to the Cell Line Ontology (CLO), including precursor cells and experimentally modified cells, were added to the ontology as needed to obtain identifiers as well; such annotations enable inclusion, by external reference, of source organ and patient data, key cell derivation citations, and disease annotations [30]. If an annotation was irrelevant or not available, it was left blank. Full annotations are provided in Supplementary Table S1, with a primary assay and the associated toxicity assay sharing a single table row (annotated by reporter gene assay Tox21 ID and compound toxicity assay, respectively), thus simplifying and clarifying the result types.

### 2.2. Data Cleaning and Aggregation of PubChem Activity Outcome Results

**Figure 1:**
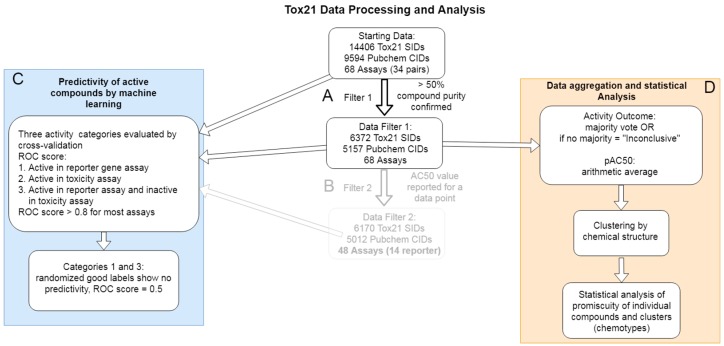
Data processing workflow. (**A**) Toxicology in the 21st Century (Tox21) project data were downloaded from PubChem and combined to a singular file for analysis. (**B**) Data were filtered based on reported sample purity and aggregated by unique compounds. (**C**) Three activity categories were benchmarked based on their chemical structures using Laplacian-corrected Naïve Baysian classification. Label randomization led to random predictions as expected. (**D**) Validated filtered and aggregated datasets were used to analyze single compound promiscuity, and scaffold promiscuity after clustering by chemical structure (**D**).

Following the retrieval of the Tox21 data from the PubChem data repository, filters were applied to refine and improve the usability and utility of the data. Due to inadequate purity, 8034 compound batches (55.7%) were removed, corresponding to a removal of 4437 PubChem CIDs (46.2%) (Figure 1A). Because not all compounds (SIDs) were tested in all assays to begin with, some subsets of the data were disproportionately reduced during this filtration step. Eleven assay pairs contained 5157 unique CIDs, 21 assay pairs contained 3354 unique CIDs, and two assay pairs contained only 62 unique CIDs, for a total matrix of 254,574 data points, or 127,287 when paired as reporter/toxicity assay sets (Supplementary Tables S2 and S3). As noted in Methods, not all SIDs in the Tox21 library were tested in every assay, thus generating this disparity of total data and an incomplete matrix (Table 1 and Supplementary Table S2). Notably, the assays with the fewest tested compounds (1366 SIDs) were the two mentioned above as lacking available annotations through Tox21—ERR845 and PGC756. Importantly, for a substantial percentage of records (substance assay pairs), no AC_50_ values were reported. The number of data points without AC_50_ values was greater than $\frac{2}{3}$(67.4%) of the total (purity filtered) data, including 20 reporter gene assays that did not include a single record with a reported AC_50_ value (Figure 1B). Because of that large proportion of data without reported AC_50_ values, we focused our data aggregation and analyses on the records filtered only for purity and after detailed metadata annotation and appropriate re-formatting (Supplementary Table S3), and based on the binary PubChem Activity Outcome reported for all data points.

We also investigated substance records not active in any assay based on the PubChem Activity Outcome metric. There were 3530 substance batches corresponding to 2843 CIDs that fell into this category. These records were kept and included in our data analysis, because these “negatives” are potentially valuable in modeling the data and can provide insight into compound toxicity or lack thereof.

PubChem Activity Outcome results were directly used as reported in PubChem. This metric had been calculated based on the categorization of sigmoidal curve behavior [31] after scoring results (PubChem Activity Score) on a scale from zero to 100, where inactive compounds scored zero, inconclusive compounds scored between one and 39, and active compounds scored between 40 and 100. Using the PubChem Activity Outcome, we found no substantial correlation between reported pAC_50_ values and the stated outcomes using the purity filtered dataset (Supplementary Figure S1). However, it should be noted that the pAC_50_ value was just one of the curve fit parameters, and the activity outcome score had been derived from the overall characteristics of the concentration response curve. To enable the differentiation of actives at very high concentrations—likely due to unspecific cell stress [13] from target-specific actives at lower concentration—data were further categorized into actives at high or low concentration. A concentration cutoff of pAC_50_ = 5.15 was established based on the distribution of the pAC_50_ values of the data points annotated as active (Supplementary Figure S2). One standard deviation above the pAC_50_ mean resulted in the cutoff of 5.15 and was used to indicate compounds active at low concentrations. Active and inactive annotations for all compounds for all assays and actives at high and low concentrations are reported in Supplementary Table S3. For the analyses below, we utilized the binary active/inactive classifications.

To gain more insight into the data (in particular, into possible mechanisms of actions of tested compounds), to enable further analysis, and for re-use of the data by others, we aggregated the data and then considered three categories of active compounds based on the annotations of the assays. First, for each assay (AID), PubChem Activity Outcomes were aggregated by unique compounds (CID) as described in Methods (and shown above in the numbers of unique CIDs). Using these aggregate activity outcomes and the curated assay annotations, we considered the following categories of active compounds: (i) compounds active in one or more reporter gene assays, (ii) compounds active in one or more cell viability (toxicity counter screening), and (iii) compounds active in a reporter gene assay, but inactive in the corresponding cell viability (toxicity counter screening) assay. It should be noted that compounds found active in the first category likely include artifacts related to the assay technology (reporter system) and possibly toxicity. Artifacts related to the assay technology would also be expected in the second category. The third category of reporter actives but counter assay inactives can be considered reporter selective. We would also expect this classification to remove generally toxic compounds, including these that induce cell stress at high concentrations. To simplify these categories for this report, accordingly, we term the three categories (i) reporter assay active, (ii) toxicity assay active, (iii) reporter assay selective. To make these datasets readily reusable, we provide the aggregated signature-level datasets for each category separately in Supplementary Tables S4–S6, respectively. These tables indicate which CIDs fit into the individual category for each assay and are arranged into full (dense) data matrices.

### 2.3. Benchmarking of Aggregate Tox21 Dataset by Machine Learning and Cross Validation

**Figure 2:**
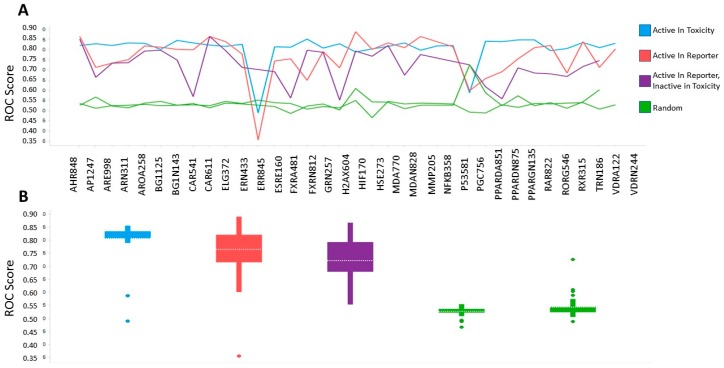
Benchmarking Machine Learning Results. (**A**) Compounds were evaluated for active versus inactive class in three distinct categories (see text for details) based on their chemical structures. Receiver operating characteristic (ROC) scores were calculated based on leave one out cross validation. Results for randomized labels are shown in green. (**B**) Box plots indicate overall ROC score distribution for each category; arithmetic means are indicated by dashed lines; error bars = standard deviation.

To statistically evaluate the quality or correctness of the aggregate activity outcome results, we needed to benchmark the results using a generally accepted reference set. While numerous studies have utilized the Tox21 data previously [5,6,7,8,9,10,11,12,13,14,15], they primarily focused on one or two specific assays or sets of primary and toxicity screens. We wanted to perform an unbiased benchmark, and in the absence of a comprehensive independent and unbiased reference dataset, we elected to use the chemical structures as an uncontroversial “ground truth” reference. In a high quality dataset, we expected the results to relate to—and therefore be predictable based on—the chemical structure of the tested small molecule. We built Laplacian-corrected Naive Bayesian machine learning classifiers as described in Methods. This method appeared appropriate for benchmarking as a frequentist approach to model the relationship of chemical features and “activity” while adjusting for the different sampling frequencies to avoid overrepresentation of rare features. Models were trained for each assay using the aggregate activity outcome of the three categories—reporter assay active, toxicity assay active, or reporter assay selective—as the definition of active. For each category in each assay, compounds labeled “active” were used as the “good” (active) class, and all other compounds were considered inactive. As descriptors of the chemical structures, ALogP, molecular weight, number of hydrogen bond donors and acceptors, number of rotatable bonds, fractional polar surface area, and ECFP6 extended connectivity fingerprints [32] were used. The models were evaluated based on the area under the receiver operating characteristics (ROC) scores obtained by cross validation. All model statistics are provided in Supplementary Table S7. As noted above and in Table 1, not all assay pairings contained the same number of CIDs on which to build benchmarking models, most notably ERR845 and PGC756, each only containing 63 unique CIDs. To confirm that the algorithm indeed learned the activity outcome based on the chemical structure descriptors, the active and inactive labels were randomized while maintaining the fraction of actives. Figure 2 shows the ROC scores for the three categories along with the randomized label controls for a total of 162 models (98 actual and 64 randomized). For 49 of the classifiers based on the three activity categories, ROC scores were greater than 0.8, and for a further 18 ROC, they were greater than 0.75. These models also showed good enrichment of actives at 1%, 5%, and 10% sampled fractions, further supporting good predictivity of these models. ROC scores of the randomized labels classifiers were close to 0.5 for most models, as expected. These results confirm that the activity categories of the compounds are statistically related to their chemical structures, and that the PubChem Activity Outcome has sufficient signal to distinguish active and inactive compounds. Interestingly, activity in the toxicity assay active category resulted in the lowest overall variance and the highest average ROC score (0.82) among the criteria evaluated, suggesting that chemical structure may serve better to predict activity in the general cell viability assays compared to the reporter gene assay activity (Figure 2B).

### 2.4. Analysis of Compound Promiscuity in the Tox21 Dataset

**Figure 3:**
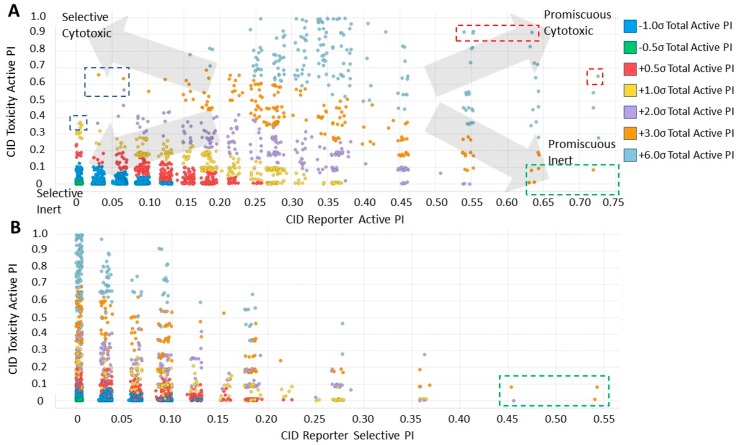
Promiscuity-based classification of individual compounds. (**A**) Reporter activity PI relative to toxicity activity PIs classifies compounds into groups of selective versus promiscuous (measured by relative reporter activity PI) and inert versus cytotoxic (measured by relative toxicity activity PI). Selected members of the selective cytotoxic (blue boxes), promiscuous cytotoxic (red boxes), and promiscuous inert (green box) groups were examined in more depth (Table 2). (**B**) Reporter selective PIs plotted relative to toxicity activity PIs. Examples of CIDs with high reporter selective PIs and low toxicity PIs (green box) were examined in-depth (Table 2). Data points are colored by total activity PI z-scores indicative of general promiscuity and were jittered on x- and y-axes for clarity due to non-continuous values of the PI values.

To gain more insight into potentially specific or nonspecific actions of compounds, we investigated their promiscuity in the three activity categories described above. Utilizing the PubChem Activity Outcomes reported, we quantified promiscuity (as indicated by a higher proportion of “Active” annotations) in the categories tested by calculating simple promiscuity indices (PI) for each category (see Methods). PIs of individual compounds and chemotype clusters were transformed into z-score (standard deviations from the mean) (Supplementary Figure S2). Using the promiscuity z-score, 465 CIDs were considered potentially biologically privileged or specific to an important regulator hub, because they were only promiscuous (z-score ≥ 1) in the reporter selective category (Supplementary Table S6). These were active in several reporter assays, but not in their corresponding counter assays, suggesting that they mechanistically acted on the reporter gene pathway. Additionally, 806 compounds were found to be generally promiscuous based on their PI across reporter and toxicity assays (z-score ≥ 1). Of them, 24 compounds were considered potentially cytotoxic (toxicity PI z-score ≥ 1), and 207 were considered reporter promiscuous compounds (reporter assay PI z-score ≥ 1). The preprocessed data were examined by plotting PIs of reporter active versus toxicity active. Separating the compounds into quadrants suggested these general categories: high indices in both reporter and toxicity assays, high reporter index and low toxicity index, low reporter index and high toxicity index, and low indices for both reporter and toxicity (Figure 3A). In addition, we examined example compounds for three of the quadrants with reporter PI/toxicity PI ratios as quadrant representatives: ~0.7 (promiscuous cytotoxic), ~8 (promiscuous inert), and ~0.1 (selective cytotoxic) (Table 2, Figure 3A), as well as compounds known to interfere with the reporter fluorescent proteins. Examples of compounds with low reporter PI and moderate to high toxicity PI values (selective cytotoxic group in Figure 3A) included diverse chemical structures such as the anticholinergic tertiary amine oxybutynin (CID 91505) and the hydroxamic acid broad-spectrum matrix metalloprotease (MMP) inhibitor Ilomastat (CID 132519). More cytotoxic compounds included industrial surfactants, a long alkyl derivatized benzene sulfonic acid (CID 29249), and a long alkyl primary amine (lauryl amine; CID 458426).

Compounds with high PIs for both reporter activity and toxicity activity (promiscuous cytotoxic group in Figure 3A) included several extended electron-deficient aromatic systems, including cyanine dyes [(Carbocyanine/Quinaldine Blue (CID 5709754), C3-thiacarbocyanine (CID 16211385), dithiazanine iodide (CID 10578)], an anti-infective bis(nitrofuryl-vinyl)-allylideneamino guanidine (CID 13118), as well as Idarubicin (CID 636362), a topoisomerase II inhibitor.

Compounds with high reporter PI and low toxicity PI values (promiscuous inert group in Figure 3A) included a benzodiazeopine (CID 443375); benzodiazepines are privileged scaffold, supporting the idea that these may act on different reporters. Further compounds included Lercanidipine (CID 157917), a calcium channel blocker antihypertensive, and Phanquinone (CID 6764), a reactive (oxidizing agent) antiparasitic and insecticide. We also performed a similar analysis for PI of reporter selective compounds (Figure 3B). These compounds had a high reporter selective PI/toxicity activity PI ratio (≥5), indicating high selectivity of reporter activity but inert for toxicity. This was a similar category to those in the promiscuous inert group above that included some overlap but were statistically significant for reporter activity and toxicity inactivity. They included multiple receptor binders, including Devazepide (CID 443375), described above, Dinaline (CID 42725), an antineoplastic agent, and Broquinaldol (CID 65620), an antifungal and antibacterial.

### 2.5. Cluster Promiscuity and Chemotype Analysis

**Figure 4:**
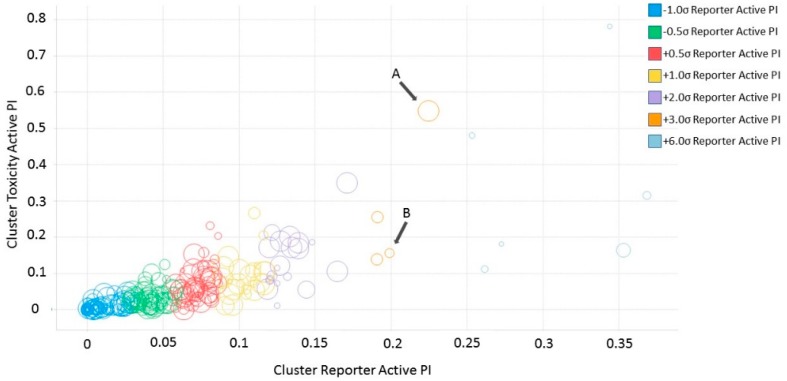
Promiscuity-based classification of chemotype clusters. Similar to Figure 3 for individual compounds, chemotype clusters were visualized based on reporter activity PIsand toxicity activity PIs. Each cluster is represented by one data point sized by number of members within the cluster and colored by total activity PI z-scores. Most clusters resulted as specific and inert (lower left corner). Two clusters, designated A and B, were examined further because of cluster size and high reporter/toxicity PI ratio (A), and molecular scaffold similarity to luciferase inhibitors (B).

To evaluate promiscuity of chemotypes, the compounds in the filtered dataset were clustered by their chemical structure topology as described in Methods; 258 clusters with an average of 20 members were obtained. As with individual CIDs, compound clusters were analyzed for promiscuity, calculating PIs for reporter active, toxicity active, and reporter selective categories, in addition to a general (total across all assays) promiscuity index of the cluster. Following the same procedure and categories for CIDs, z-scores of PIs were determined by standard deviation from the PI mean. Using z-scores in the respective categories, 612 CIDs corresponding to 23 clusters resulted as potentially biologically privileged or specific (reporter selective PI z-score ≥ 1). There were 49 clusters corresponding to 1241 compounds found to be generally promiscuous (total activity PI z-score ≥ 1). Three clusters corresponding to 26 compounds were found to be generally cytotoxic (toxicity PI z-score ≥ 1). Finally, 16 clusters corresponding to 294 compounds were found to be promiscuous in reporter assays (reporter active z-score ≥ 1). While most clusters were found to be selective and inert (Figure 4), we further examined two clusters, 53 and 251 (Figure 4A,B, Table 2). Cluster 53 (Figure 4B) was chosen due to the presence of a known luciferase inhibitor, Ataluren (CID 11219835) [33], suggesting an assay artifact. Cluster 251 (Figure 4A) was chosen due to its high toxicity active PI of 0.54, relatively high reporter active PI/toxicity active PI ratio of 0.41, and large size (22 molecules). Other clusters of similar or higher ratio values only contained one to five compounds. We can hypothesize that cluster 53 (Figure 4B), containing the luciferase interfering compound Ataluren, included other luciferase inhibitors or stabilizers as well. Ataluren has been shown to stabilize luciferin by formation of an AMP mixed anhydride [34] by binding the Ataluren carboxylic acid group. Of the seven compounds in cluster 53, two others (CIDs 68706 and 3059) contained similar structures of carboxylic acids attached to rings, indicating a similar luciferase inhibition mechanism could be occurring, though there is no evidence of this currently, and both compounds had very low reporter activity PI values. Interestingly, Ataluren and CID 3059 shared similar PI z-score results, with both having a z-score of +2 for reporter selectivity. While Ataluren’s luciferin-binding structure was not common throughout, five of the seven compounds in the cluster shared Ataluren’s significance in privileged, reporter active, and total active z-scores, potentially interfering via anther mechanisms. The majority of compounds (20 of 22) of the second cluster, 251, were long-chain cationic surfactant-like molecules (particularly quaternary ammonium and phosphate salts) (Table 2), a likely mechanism for the noted increased toxicity due to the germicidal nature of the structures [35]. The remaining compounds within cluster 251 were CID 5374, an adrenergic receptor and dopamine agonist, Talipexole [36], and CID 16213711, 1-butyl-1-methylpyrrolidinium dicyanamide, a hypergolic ionic solute granting a large electrochemical potential range [37,38]. Twelve members of cluster 251 had a very high PI z-score for toxicity (≥6); they were all surfactants, with one having a z-score of three. Full information for CID clustering, including SMILES and aggregated Tox21 structure IDs (SIDs), are provided in Supplementary Table S8.

## 3. Discussion

Advances in screening technologies, including detection sensitivity and throughput, robotics, and data science, have enabled many large scale data generation projects during the last two decades [26,30]. Examples of publicly funded research consortia focused on small molecule discovery and characterization include the Molecular Libraries Program (MLP) [39], the Tox21 screening program, the Psychoactive Drug Screening Program (PDSP) [40], the Library of Integrated Network-based Cellular Signatures (LINCS) [21], and Illuminating the Druggable Genome (IDG) [41,42]. While such individual projects can have enormous scientific impact, their combined value and impact may yet be considerably larger, because integrated “big data” have potential to provide new insights that cannot be obtained from individual datasets. However, the value of big data is difficult to unlock, as many screening datasets are nearly impossible to navigate for the uninitiated user [43]. The many challenges of data integration and reuse include adequate data reporting standards, deep metadata annotations, consistent, globally unique, and resolvable IDs, reproducible data processing pipelines, open data formats, documented protocols for data access, etc. In part, the FAIR guiding principles for scientific data management and stewardship [16] seek to remedy several of these key issues facing data users. For the purposes of this current work, interoperability and reusability are the aspects of Tox21 we sought to address. By enhancing the definition and annotation of Tox21 assays using controlled vocabulary resources, users will be better able to determine which, if any, assays are relevant to their individual integrative analyses. Knowledge and easy accessibility of vital integrative and descriptive features, such as parental cell line/species, affected gene or pathway, reporter type, endpoint type, and assay stage of confirmatory versus Counter, will enable researchers to combine/integrate the data more readily with other data from additional sources.

As part of FAIRness issues in published data, analysis of high throughput data relies not only on access to proper annotations but knowledge of experimental procedures, methodologies, and interpretation (actionability) of the produced results. This is particularly true for users with less experience in large-scale data analyses [43]. The Tox21 datasets examined here contain > 1.8 million data points, including replicates [17]. The compiled aggregated datasets do not constitute full dense matrices, because not all compounds were tested in all assays, and some SIDs were removed due to purity. Signature-level data (Supplementary Tables S4–S6) were each separated into three dense matrices based on the number of compounds remaining following data cleaning and preprocessing, as shown in Table 1. Notably, in one subset, as few as 1366 compounds of 14,406 total were tested. In part, this limits global aggregate-based analyses, and instead, the assays were first examined pair-wise with the reporter assay and its associated toxicity assay. While large datasets can provide researchers with many valuable insights of biological function, much can be gained from a more standardized dataset with human and machine readable (actionable) results and a clean data structure. For the Tox21 datasets, we first cleaned the data by removing (filtering out) all records associated with samples of insufficient or unconfirmed purity. This was a necessary step, because screening results with degraded or otherwise contaminated compounds would not have been useful or reproducible. This simple filter removed more than 50% (8034 of 14,406) of total SIDs from the initial library. Next, we wanted to create a dataset of unique compound structure-bioactivity data points. Each assay represented a unique biological outcome, which was formally described by standardized annotations, as described above. In many cases, different compound samples (SIDs) corresponded to the same chemical compound structure, for example, if a sample was available from different suppliers, purchased twice (as different batches), or if different salt forms of a small molecule existed. PubChem, via a chemical structure standardization and registration process, associates each sample (SID) with a unique standardized chemical structure (CID). For each assay, we could therefore aggregate screening results of SIDs that corresponded to the same CID. We investigated the best result types to use. At first, the reported quantitative AC_50_ (half maximum active concentration) values appeared to be the most attractive for analysis. However, for over two thirds of the data records in the Tox21 dataset, the AC_50_ values were not reported because a curve fit of the concentration response data points could not be obtained. Filtering these data points would have completely removed (i.e., all data from) over 200 SIDs and 20 assays. Moreover, using the reported AC_50_ values, there was no clear correlation of the reported activity outcome (active, inactive, inconclusive) to the pAC_50_ value (Supplementary Figure S1), in part because the AC_50_ concentration is only one parameter of a compound’s biological activity. We therefore opted to use the categorical PubChem Activity Outcome, which was generated by the Tox21 data producers after substantial research of curve classification (see above). The activity outcome results were aggregated for each unique compound and assay pair, as described in Methods. We also annotated activities at high concentrations versus low concentrations as additional criteria to enable differentiation of potential general stress-induced activity versus likely selective action on the reporter pathway (Supplementary Table S3 and Supplementary Figure S2). The main aggregated activity outcome results were then classified based on the assay annotations as reporter active or (cyto)toxicity active and an additional category, reporter selective, generated by combining results from each reporter gene assay with its corresponding counter assay, e.g., active in the reporter gene assay and inactive in the counter assay.

To evaluate if these results were useful, we benchmarked these aggregated datasets. Ideally, one would use a gold standard reference dataset for benchmarking. While it may be feasible to use external datasets, such as the LINCS small molecule L1000 transcriptional signatures [18,44], additional target-based screening datasets in PubChem, or molecule activity data in ChEMBL, these external datasets do not include all Tox21 compounds, and they may not be a generally accepted reference gold standard. We therefore used the chemical structures as an uncontroversial “ground truth” to evaluate if the aggregated results could be predicted based on the chemical structure information alone. If the aggregated results were high quality, they should have statistically been related to the chemical structure of the tested small molecule, i.e., more similar chemical structures should have more similar bioactivity outcomes compared to less similar compounds. To test this in the most general way, we built Laplacian-corrected Naive Bayesian classifiers for each individual assay (reporter active and toxicity active) and the aggregated reporter selective categories, as well as randomized actives. These classifiers were then evaluated by cross validation, and ROC scores were computed. For the vast majority of the results, ROC scores were greater than 0.75, and for half of the datasets, they were greater than 0.8. Randomizing the activity labels resulted in ROC scores of 0.5, as expected, and confirmation that the structure-activity relationships (and not some other patterns) were learned (Figure 2 and Supplementary Table S7). These results suggest good quality of the vast majority of the results. Notably, the poorest performance of benchmarking, from assay ID ERR845, was one of two assay pairs with far fewer tested compounds (Table 1 and Supplementary Table S2), thus resulting in much less robust models. Additionally, the largest outlier of the randomized models belonged to the second assay with very few compounds, PGC756. Though randomized ROC scores were averaged over 10 repetitions, these results also suggest that our benchmarking for datasets with very few compounds was not reliable. The (cyto)toxicity active category resulted in the highest average ROC scores and the lowest overall variance, followed by reporter active and reporter selective (Figure 2B). The lower predictivity (ROC score ~0.73) of the reporter selective category outcomes could be rationalized by its aggregation from two assay categories results (reporter gene active and toxicity counter inactive), thus adding their random errors.

Complementary to the statistical evaluation of the assay result categories across all compounds, one can analyze the results for each individual compound or chemotypes across relevant subsets of assays. One such measure is a simple PI, defined as the ratio of active versus total (tested) assays of a category of interest for any one compound or chemotype. The PI for different assay categories defined by their standardized annotations can reveal distinct activity patterns and facilitate hypotheses of possible mechanisms of promiscuity or selectivity. While broadly promiscuous compounds are mostly undesirable, many of the Tox21 reporter genes pathways shared functional similarity. It could therefore be expected that some compounds were active in several reporters. For example, numerous reporter promiscuous compounds had chemical scaffolds similar to steroidal hormones (Supplementary Table S4). While the propensity of high promiscuity for these compounds is relatively well known, it is particularly important to note that 13 of the 34 (reporter and toxicity counter) assay pairs examined hormone pathway and receptor activity. It therefore stands to reason that steroidal hormone agonists would be somewhat promiscuous throughout the combined dataset. Additionally, with 12 of the 34 assay pairs utilizing a luciferase based reporter system, they were vulnerable to interference by compounds including Ataluren (CID 11219835 [33]). Examining Ataluren’s cluster (cluster 53; Table 2) revealed that these potential luciferase inhibitors, while promiscuous (likely assay artifacts), did not have very high PI values overall. Additionally, structural examination revealed two CIDs within cluster 53 potentially able to bind luciferin as Ataluren does [34], one of which had highly similar PI results to Ataluren. Low PI values may have been due to the remaining 22 assay pairs utilizing a beta lactamase based reporter system.

Subsets of promiscuous molecules could be further analyzed by their chemical structures, facilitating explanations or hypotheses of their mechanisms leading to promiscuity (Table 2). Compounds with high toxicity PIs revealed chemical structures of known cell toxicity, including long-alkyl chain cationic and anionic surfactants such as lauryl amine or dodecyl-benzenesulphonic acid and derivatives. Compounds with high PIs for both the reporter and toxicity assay activities had a high proportion of extended electron deficient aromatic systems, including cyanine dyes. They also included DNA intercalators such as idarubicin, which are generally toxic and used and chemotherapeutics. Compounds with high reporter and low cyto(toxicity) promiscuity included several targeted therapeutics, including a Devazepide, a benzodiazepine CCKA receptor antagonist, the beta blocker Lercanidipine, and the antiprotozoal Phanquinone. These examples illustrate several meaningful activity categories across the Tox21 dataset. In many cases, they could easily be related to their chemical structures, chemical reactivity, or known mechanism of action. Together with the machine learning-based benchmarking, these results are suggestive of a high-quality reusable dataset with interpretable results. The aggregate highly annotated dataset can be integrated with other data and is reusable, for example, to make predictions of the tested compounds or as a reference profiling dataset. We are currently investigating how the aggregated and cleaned Tox21 profiles can be best integrated with small molecule perturbation-response signatures from the LINCS project. We are also investigating if and how this data can be integrated with the IDG project. To facilitate FAIRness, we made the annotated, cleaned, and aggregate Tox21 dataset available via the LINCS Data Portal [18] (Aggregated Tox21 bioactivity data, 2019) [28].

## 4. Materials and Methods

### 4.1. Tox21 Dataset Retrieval and Annotation

The Tox21 project collective data, as listed on the official data browser [17] consists of 47 primary assays, 34 of which had been published with a corresponding counter assay at the time of this analysis. While Tox21 and the EPA have released more assay data through sources including ToxCast [29], for this study, we sought to focus on these major primary/confirmatory assay pairs provided by the Tripod website. In order to facilitate computational analysis and improve FAIR compliance, the datasets were annotated primarily through the use of controlled vocabulary based on classes from the BioAssay Ontology [23,24,25,26,45]. The most important annotations included assay title, reporter gene assay (ID of the reporter assay), reporter gene assay (indicating the type of reporter utilized), cell line cell (indicating the initial cell line utilized for assays prior to reporter modifications), and target relationship (indicating if the assay sought target agonists or antagonists). In addition, fields not included in BAO but which are both human and machine readable, were included, such as Uniprot/gene ID of the target, the organism of the cells (including NCBI taxonomic ID), and reporter gene assay Tox21 ID. We also included Gene Ontology (GO) [46,47] annotations of the primary pathway affected as target annotations. Annotations of the assays were performed manually as a data curation step by examining each assay description in detail. Supplementary Table S1 summarizes the assay annotations. Importantly, two of the reporter/counter screen pairings had notably incomplete annotations (reporter assay IDs ERR845 and PGC756). No cell line was identified as a precursor; instead, they were identified as the ERR line and the PGC/ERR line, respectively. Neither assay description was available via the Tox21 tripod site [48], and most annotations in Supplementary Table S1 were obtained through the individual PubChem assay pages.

Data for the 68 assays (34 primary and 34 counter) were retrieved through the PubChem API bioassay tools via Pipeline Pilot 2018 v18.1 (BIOVIA, San Diego, CA, USA). Retrieved data included for each PubChem SID and assay the PubChem Activity Score and Outcome reported as “active”, “inactive”, or “inconclusive”, which had been assigned based on the sigmoidal curve shape and fitting results [29]. Data were downloaded as individual delimited text files and mapped to the curated assay annotations by their respective PubChem assay IDs (AID). The individual files were then combined into one data file containing the results for all assays along with assay-specific annotations. Importantly, not all datasets utilized the full library of Tox21 molecules, with two sets of reporter/toxicity assays using only 1366 of the 14,406 total SIDs available, significantly reducing available data for further analysis. This difference in tested molecules was due in part to the mailability of the Tox21 library, including adding and removing samples throughout the time of data collection.

In addition to the primary datasets associated with the Tox21 project, additional metadata was downloaded from the project browser site associated with the compound batches utilized [49], including internal Tox21 IDs, quality control data, and PubChem structure CIDs. CIDs, SMILES strings, and calculated properties for each Tox21 sample were obtained via the PubChem API in order to enable data aggregation and cluster analysis. SIDs for which no PubChem CID was assigned were removed from the dataset and were not included in the analyses.

### 4.2. Data Cleaning

Downloaded data were further filtered and processed using Pipeline Pilot 2018 v18.1 (BIOVIA) to obtain a consensus quality dataset from the Tox21 project. Samples were first filtered based on their purity to remove low quality data records (Figure 1A). Based on sample data from Tox21, individual SIDs were filtered on the results of the two quality test timepoints, T0 and T4, irrespective of the methodology used to establish the results. In order to ensure the most accurate data, we removed samples that did not have either an A (≥90% purity and molecular weight confirmed), B (75–90% purity and molecular weight confirmed), or C (50–75% purity and molecular weight confirmed) rating in T0 and either an A, B, C, or untested rating result from the T4 examination.

We considered another filter (Figure 1B) in which we examined records with and without reported AC_50_ values. For 202 SIDs, no single AC_50_ value was reported in any assay. Further, 20 reporter assays did not contain any SID record with a reported AC_50_ value. Were we to consider only records with reported AC_50_ values, 202 samples and 20 assays would thus have been removed entirely from the dataset. In fact, as shown in Figure 1B, for most AID-SID records, no AC_50_ value was reported. We therefore focused on PubChem Activity Outcome to create a consensus aggregate dataset and for further analysis in which we did not remove the records with missing AC_50_ values, instead utilizing only the purity filtered dataset (Figure 1A).

To obtain activity records for each unique chemical compound, the reported PubChem substance (SID) associated activity results for each individual assay across the entire Tox21 dataset were aggregated by CID (Figure 1D). Not all SIDs had an assigned CID; these records were removed from the dataset and were not included in any of the analyses reported here. Sample batch replicate p AC_50_ (−log_10_(AC_50_)) values were averaged (by SID) when present per assay. PubChem activity outcome results of active, inactive, or inconclusive were presented as majority, i.e., if >50% of SIDs showed a common outcome, it was reported as such. Any aggregated activity outcome results with no majority were changed to inconclusive. This aggregate dataset was intended for further analysis such as the collective effects and potential regulatory pathway specificity of individual molecular structures and chemotypes. Additionally, the data were aggregated by CID at each filtration step described above for the purpose of examining how many unique compounds remained through each filter.

Because for the majority of AID-CID data points, no AC_50_ results were reported (Figure 1B), we used the purity filtered (Figure 1A) datasets and their reported aggregate PubChem Activity Outcome results as our main Tox21 aggregate dataset for further analysis outlined below. In addition, activity at high concentration versus low concentration was annotated to enable differentiation of potentially general stress-induced activity versus likely selective action on the reporter pathway. A pAC_50_ cutoff of 5.15 was established based on the distribution of the pAC_50_ values of the data points annotated as active (Supplementary Figure S2). The pAC_50_ cutoff of 5.15 corresponded to the mean plus one standard deviation, e.g., z-score = 1. The activity annotations for all compounds and all assays are provided in Supplementary Table S3.

The numbers of compounds with corresponding assays and unique data points of the cleaned datasets are provided in Supplementary Table S2. Each subset represented a distinct, dense matrix of the number of unique number of compounds times the number of assays due to the changes in the Tox21 compound library during data collection. While the 3345 and 64 compounds in subsets two and three were included in the 5157 compounds of subset one, the assays were different, and the data points of the subsets did not overlap. Signature level data were separated into these subset matrices, as shown in Supplementary Tables S4–S6.

Filtered data were presented in two primary forms: (i) a table containing CIDs and their corresponding pAC_50_ values and activity outcome results for every reporter/toxicity assay pairing (Supplementary Table S3) and (ii) as separated binary signature tables representing reporter active, toxicity active, and reporter selective annotations for each CID per assay, where one indicates a positive result and zero represents negative for the categorization (Supplementary Tables S4–S6).

We made these cleaned datasets publicly available as supporting information and via the LINCS Data Portal [18] as dataset group EDG-1016 (Aggregated Tox21 bioactivity data, 2019) [28]

In addition to the results by unique chemicals for each individual assay (AID), we also aggregated PubChem Activity Outcome results across reporter gene and their corresponding cell viability (toxicity) counter assays using the BAO annotations described above. Specifically, we defined compounds “active” in a reporter gene assay but “inactive” in the corresponding cell viability (toxicity counter screening) assay. With that, we created three main categories of “active” compounds: (i) “reporter assay active”, those that are active in one or more reporter gene assays, (ii) “toxicity assay active”, those that are active in one or more cell viability (or toxicity) assays, and (iii) “reporter assay selective”, those that are active in a reporter gene assay and inactive in its corresponding cell viability (or toxicity) counter assay (Figure 1). Importantly, signature-level data consisting of aggregated CID data in a binary yes/no affirmation of presence within the above categories by assay are presented in Supplementary Tables S4–S6. These data tables are best for reuse of the information presented here, suitable for machine learning applications, integrative analyses, and general interpretation.

### 4.3. Machine Learning Classifiers and Cross Validation

To examine the quality of the aggregate Activity Outcome Results, we benchmarked the results using their associated chemical structures. We built Laplacian-corrected Naive Bayesian classifiers for each assay and for cross assay aggregate results, as described above (Figure 1C). In all, three categories of models were created, (i) reporter assay active, (ii) toxicity assay active, and (iii) reporter assay selective. For each model in each category, a chemical with the aggregate PubChem activity outcomes relating to the category name was considered good (i.e., active class), and all other molecules were used as decoy (inactive class). Bayesian classification is computationally efficient and has been shown in many studies to be robust and predictive. A Naïve Bayesian classifier predicts active compounds based on the frequency of occurrence of chemical features in a training set of active and inactive compounds. The Laplacian correction accounts for the different sampling frequencies of the chemical features assuming that most features have no relation to activity. Models were built in Pipeline Pilot 2018 v18.1 (BIOVIA). To build the classifiers, the following descriptors of the chemicals were used: ALogP, molecular weight, number of hydrogen bond donors and acceptors, number of rotatable bonds, fractional polar surface area, and ECFP6 extended connectivity fingerprints [31]. The built-in cross validation was used, and the area under the ROC score was reported. The model statistics are provided in Supplementary Table S7.

To verify that the models did indeed benchmark the aggregate activity categories based on chemical structures, classifiers were built using the same procedure, except randomizing the active and decoy (inactive) labels. In these cases, ROC scores of about 0.5 were obtained for all tested models, as expected. Results of these analyses are shown in Figure 2 and Supplementary Table S7.

### 4.4. Compound Structure Clustering

Small molecules that were part of the aggregate purity-filtered dataset described above were clustered based on their chemical structure topology using the Pipeline Pilot 2018 v18.1 (BIOVIA) relocation algorithm based on maximal dissimilarity. The Tanimoto metric based on functional group cyclic fingerprints of length 6 (FCFP6) with an average cluster size of 20 molecules was used. A cluster is considered a chemotype.

### 4.5. Classification of Promiscuity for Individual Compounds and Chemotypes

With more refined and extensively annotated data, we sought to determine what, if any, molecules and chemotypes (compound clusters) were promiscuously active based on the varying criteria of activity above. We examined the activity of CIDs from purity filtered data based on the categorizations outlined above—reporter active, toxicity active, and reporter selective. Additionally, CIDs were examined for general promiscuous activity within the combination of both reporter and toxicity assays. Activity PI values were calculated for each compound (CID) as reported previously [50], by dividing the total number of active records (assays) by the total number of tested records (assays), and was performed for the above categories.

Activity fractions were analyzed in Tibco Spotfire Analyst v 7.11.1.0.26 (Tibco Software, Palo Alto, CA, USA), and promiscuity indices were binned based on z-score (standard deviations from the mean), with z-score ≥ +1 being recognized as promiscuous. Sample binning for reporter active PI is shown in Supplementary Figure S3. Compounds could then be classified based on their promiscuity across all assays or the “reporter active”, “toxicity active”, and “reporter selective” categories in the following sequence: (1) significant (z-score ≥ +1) total activity across all assays indicating general promiscuity, (2) significant toxicity assay activity, indicating potential cytotoxicity, (3) significant reporter assay activity, indicating potential reporter system interaction and/or biological promiscuity, and (4) significant selective activity, potentially indicating specific interaction at a major regulator affecting many reporters or some privileged biological activity interacting with several targets, but not a generally toxic compound or assay artifact.

Chemotype promiscuity was defined similarly but considering the fraction of active records (or assays) across all compounds of that chemotype (obtained after clustering of compounds based on chemical structures, see above). This corresponded to the average promiscuity index of all compounds in a cluster. As for individual promiscuity, the total active annotations across all assays (including reporter gene and toxicity) in addition to the reporter active, toxicity active, and reporter selective categories were examined.

In addition, compounds could be sub-classified based on their promiscuity (or selectivity) in the reporter active and the toxicity active categories (Figure 3). These sub-categories corresponded to the four quadrants in Figure 3 ranking compounds by PI in the reporter active and the toxicity active categories; they were termed promiscuous inert, promiscuous cytotoxic, selective inert, and selective cytotoxic. A PI of 0.45 on the reporter active fraction and 0.3 on the cytotoxicity active fraction was chosen to bias the promiscuous quadrants towards the highest promiscuity z-score (≥+6; teal, Figure 3). A small number of examples at the edges of each quadrant, annotated by dashed boxes in Figure 3A, were selected for an illustrative in-depth analysis. A similar analysis was performed considering the reporter selective versus the toxicity active fractions (Figure 3B). The most extreme examples of selective inert compounds, designated by the dashed boxes, were further analyzed.

Similarly to individual CIDs, molecular clusters (chemotypes) were plotted based on fractions of reporter active and cytotoxicity active assays (Figure 4) while also indicating cluster size. Two clusters were selected for closer investigation—cluster 251 (Figure 4A) and cluster 53 (Figure 4B). Cluster 251 was chosen due to its large size (22 compounds) and high toxicity active fraction. Cluster 53 was chosen because it contains a known, highly potent luciferase inhibitor (CID 11219835) and because of the large number of luciferase-based reporter assays in the Tox21 project.

### 4.6. Characterization of Molecular Scaffolds and Features

Chemical structures of selected CIDs and clusters outlined above were examined manually for key structural features and functional groups to gain further insights into potential functional characteristics, such as toxic groups, DNA intercalators, and other known molecular scaffolds. Additionally, PI z-score results of the CID-based categorizations of reporter active, toxicity active, and reporter selective for the seven members of cluster 53 were examined for significance to hypothesize potential mechanisms of the cluster members. Chemical structures were obtained from PubChem based on the canonical SMILES associated with the CID.

## 5. Conclusions

The Tox21 project is an enormous undertaking with a tremendous amount of data and potential for finding new biological insights and chemical interactions. As with many large datasets, significant effort is required for data curation and interpretation to use the Tox21 data. The full utility of the dataset is limited by difficulties of findability of data and metadata, accessibility, interpretability of the reported results, internal data inconsistencies, and ultimately interoperability and reuse. In this study, we constructed a Tox21 dataset with the goal to improve its utility with respect to all FAIR criteria. We provided deep metadata annotations of the assays and the screening results and a clean and simple representation of the data as (aggregated) signatures that correspond to interpretable result categories with clear metadata and are suitable as input for computational analyses. We benchmarked the Tox21 signatures and illustrated their utility by relating them to chemical structures, reactivity, or known mechanisms of action. The combined and cleaned Tox21 dataset includes 5157 unique compounds and 68 assays. Although the cleaned dataset and final signatures are not full dense matrices, they represent highly relevant and likely very useful data for other projects. Our work highlights the need for implementing data standards and accurate detailed annotations when reporting screening results that are meant to be reused in the community. We hope that the extensively annotated, cleaned, and signature-level datasets will be useful for researchers as reference datasets to make predictions or to combine with other projects. We made this cleaned dataset and signatures publicly available as supporting information and via the LINCS Data Portal [18], as EDG-1016 (dataset citation: Aggregated Tox21 bioactivity data, 2019) [28]

## Figures and Tables

**Table 1:** Data Matrix Statistics.

Set Number	Compounds Tested	Number of Assays *	Total Data Points
**Set 1**	5157	22	113,454
**Set 2**	3354	42	140,868
**Set 3**	63	4	252
**Total Unique**	5157	68	254,574
Total Data Pairs **			127,287

* Number of assays represents total of reporter assays + toxicity assays, based on compounds remaining after filtration; ** Data pairs represents the number of data points aggregated to reporter/toxicity assay pairings which are shown in Supplementary Table S3.

**Table 2:** Examples of Promiscuous Compounds.

Compounds with High Toxicity z-Scores		
Sample compound 1	Sample compound 2	Sample compound 3
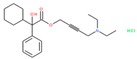 PubChem CID: 91505 Toxicity PI fraction: 0.36 Toxicity z-score: +2.0	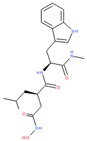 PubChem CID: 132519 Toxicity PI fraction: 0.36 Toxicity z-score: +2.0	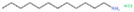 PubChem CID: 458426 Toxicity PI fraction: 0.63 Toxicity z-score: +6.0
**Compounds with High Toxicity and Reporter z-Scores**		
Sample compound 1	Sample compound 2	Sample compound 3
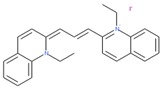 PubChem CID: 5709754 Toxicity PI fraction: 0.91 Reporter PI fraction: 0.55 Toxicity z-score: +6.0 Reporter z-score: +6.0	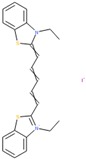 PubChem CID: 10578 Toxicity PI fraction: 0.91 Reporter PI fraction: 0.64 Toxicity z-score: +6.0 Reporter z-score: +6.0	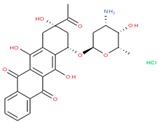 PubChem CID: 636362 Toxicity PI fraction: 0.64 Reporter PI fraction: 0.7 Toxicity z-score: +6.0 Reporter z-score: +6.0
**Compounds with High Reporter z-Scores**		
Sample compound 1	Sample compound 2	Sample compound 3
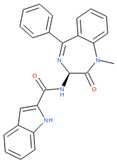 PubChem CID: 443375 Reporter PI fraction: 0.64 Reporter z-score: +6.0	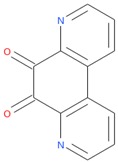 PubChem CID: 6764 Reporter PI fraction: 0.64 Reporter z-score: +6.0	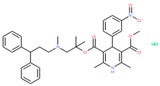 PubChem CID: 157917 Reporter PI fraction: 0.73 Reporter z-score: +6.0
**Compounds with High Reporter Selective z-Scores**		
Sample compound 1	Sample compound 2	Sample compound 3
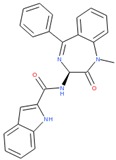 PubChem CID: 443375 Selective PI fraction: 0.56 Selective z-score: +6.0	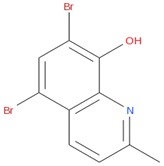 PubChem CID: 65620 Selective PI fraction: 0.56 Selective z-score: +6.0	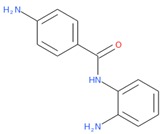 PubChem CID: 42725 Selective PI fraction: 0.46 Selective z-score: +6.0
**Compounds in Cluster 53 (Figure 4B Annotated)**		
Sample compound 1	Sample compound 2	Sample compound 3
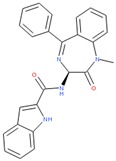 PubChem CID: 11219835 * Reporter PI fraction: 0.18 **Cluster avg. reporter PI: 0.20**	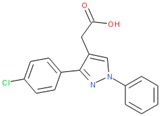 PubChem CID: 68706 Reporter PI fraction: 0.00 **Cluster avg. toxicity PI: 0.16**	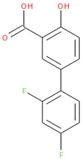 PubChem CID: 3059 Reporter PI fraction: 0.09 **Cluster avg. selective PI: 0.17**
**Compounds in Cluster 251 (Figure 4A Annotated)**		
Sample compound 1	Sample compound 2	Sample compound 3
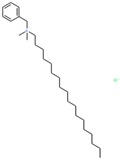 PubChem CID: 31204 Toxicity PI fraction: 0.88 **Cluster avg. reporter PI: 0.22**	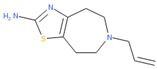 PubChem CID: 5374 Toxicity PI fraction: 0.00 **Cluster avg. toxicity PI: 0.55**	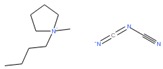 PubChem CID: 16213711 Toxicity PI fraction: 0.00 **Cluster avg. selective PI: 0.02**

* CID 11219835 identified specifically by Auld et al. [33] as a potent luciferase inhibitor. PI = promiscuity indices.

## Supplementary Materials

The following are available online at https://www.mdpi.com/1420-3049/24/8/1604/s1, Figure S1: Relation between pAC_50_ values and activity designations in unfiltered Tox21 data; Figure S2: pAC_50_ values of active compounds; Figure S3: Example promiscuity index statistical binning results; Table S1: Tox21 assay annotations; Table S2: Number of CIDs remaining per assay pairing following purity filtration; Table S3: CID aggregated pAC_50_ values for reporter gene assays and corresponding toxicity assays; Table S4: CIDs designated as reporter assay active by assay; Table S5: CIDs designated as toxicity assay active by assay; Table S6: CIDs designated as reporter selective by assay; Table S7: Full ROC statistical calculations; Table S8: Purity filtered CID clustering and SID associations for aggregation.
