# Supplementary figures and images for: Improving the Utility of the Tox21 Dataset by Deep Metadata Annotations and Constructing Reusable Benchmarked Chemical Reference Signatures
Source: Molecules. 2019 Apr 23;24(8):1604. doi: 10.3390/molecules24081604 (PMC6515292; doi:10.3390/molecules24081604)

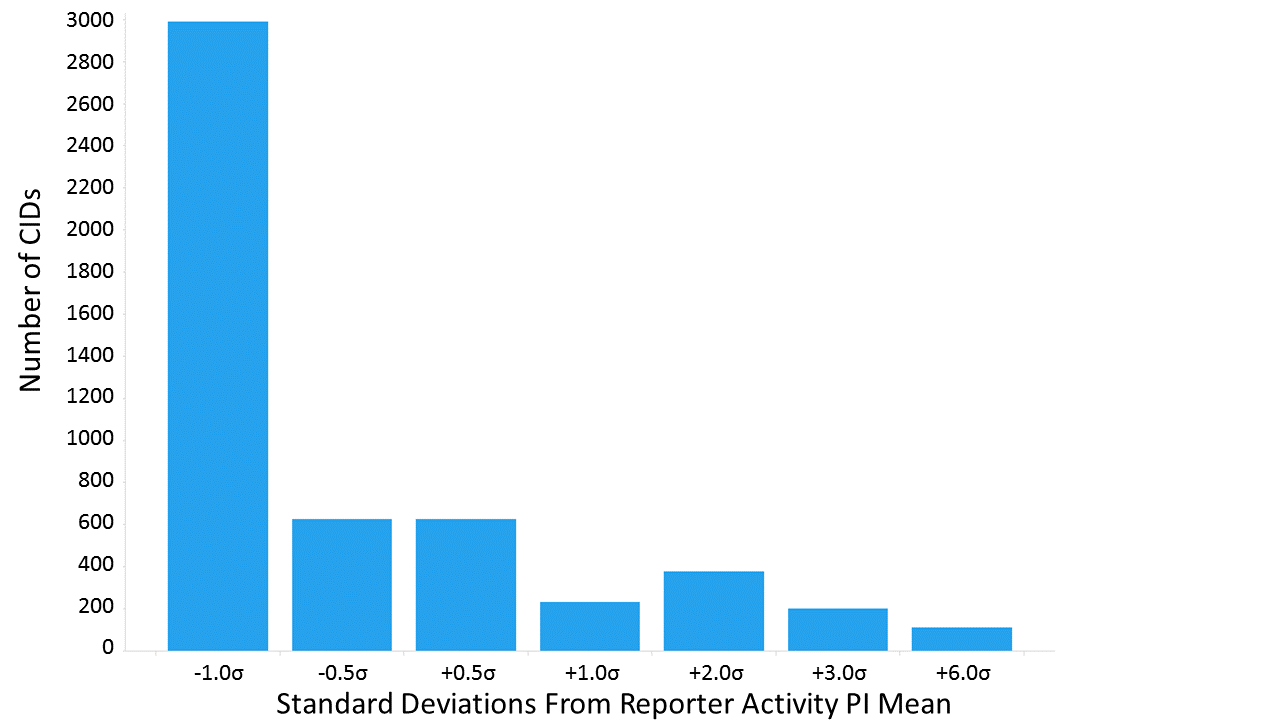

Supplement: Supplementary file 1 [file molecules-24-01604-s001.zip › Supplemental Figure 3 - Example promiscuity index statistical binning results.PNG]

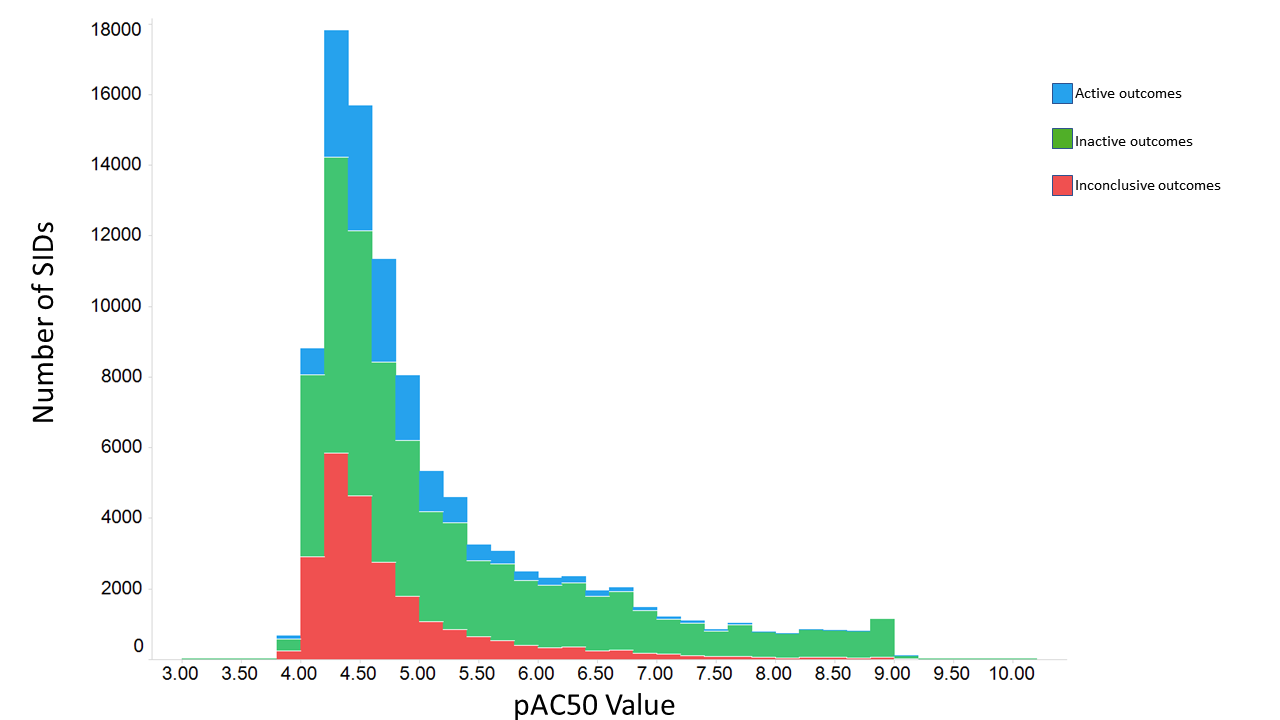

Supplement: Supplementary file 1 [file molecules-24-01604-s001.zip › Supplemental Figure 1 - Relation between pAC50 values and activity designations in unfiltered Tox21 data.png]

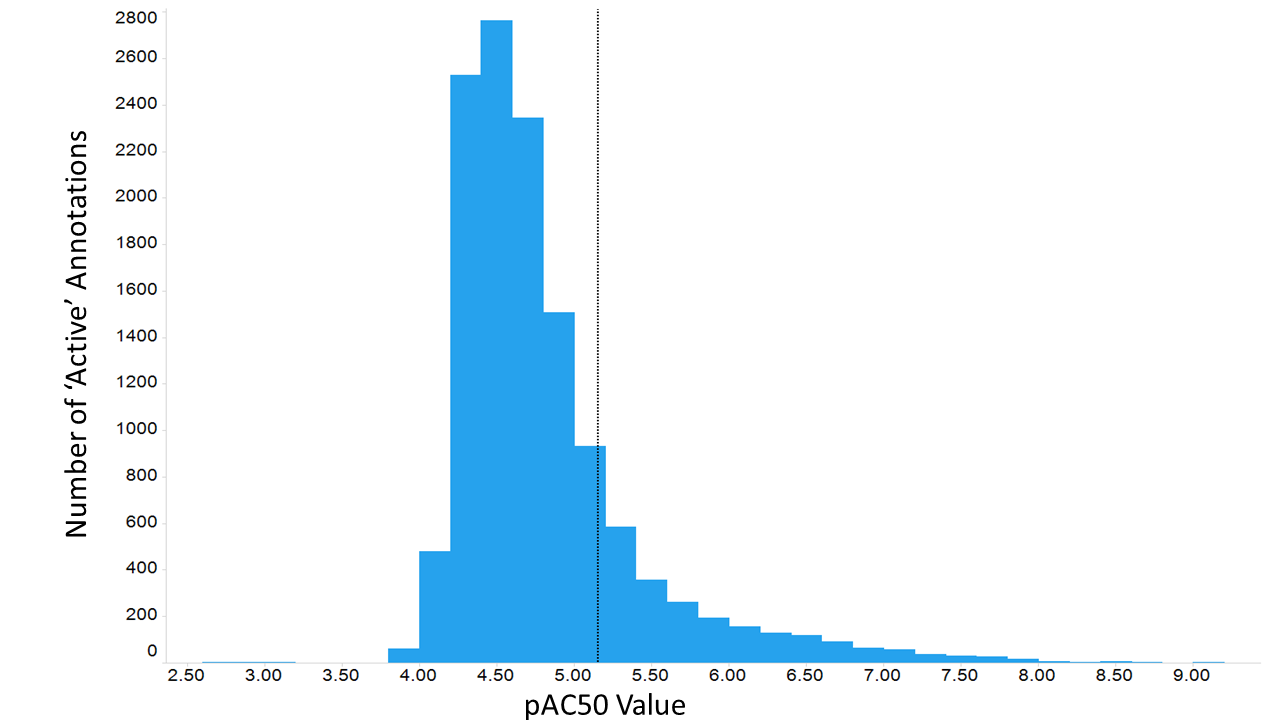

Supplement: Supplementary file 1 [file molecules-24-01604-s001.zip › Supplemental Figure 2 - pAC50 value distribution in active molecules.png]
